# *Curcuma longa* and *Boswellia serrata* extract combination for hand osteoarthritis: an open-label pre-post trial

**DOI:** 10.1080/13880209.2022.2147550

**Published:** 2022-11-23

**Authors:** Yves Henrotin, Yvan Dierckxsens, Gaëlle Delisse, Nathalie Maes, Adelin Albert

**Affiliations:** a Musculoskeletal Innovative Research Lab (mSKIL), Arthropôle Liège, Center for Interdisciplinary Research on Medicines (CIRM), University of Liège, CHU Sart-Tilman, Liège, Belgium; b Physical Therapy and Rehabilitation Department, Princess Paola Hospital, Marche-en-Famenne, Belgium; c Tilman SA, Baillonville, Belgium; d Center of Biostatistics (B-STAT), University Hospital of Liège, Liège, Belgium; e Department of Public Health, University of Liège, Liège, Belgium

**Keywords:** Curcumin, CURTIL02, boswellic acids, clinical study

## Abstract

**Context:**

Osteoarthritis (OA) of the hand is a common painful musculoskeletal disorder with no cure. There is a need for an efficient and safe treatment to relieve OA pain.

**Objective:**

To investigate the effects of a *Curcuma longa* and *Boswellia serrata* food supplement in addition to standard care on hand pain.

**Materials and methods:**

This open-label, non-controlled, post-observational study was based on 232 patients suffering from hand pain with or without joint deformity. Patients received a medical prescription for a three-month treatment with a food supplement containing 89 mg of *C. longa* dry extract, 120 mg of *B. serrata* resin, and 1.8 µg vitamin D. Pain was evaluated on a 10-point visual analog scale (VAS). The number of painful hand joints, patient satisfaction, nonsteroidal anti-inflammatory drugs intake, and side effects were also recorded.

**Results:**

Baseline pain intensity (regression coefficient ± *SE*: −0.19 ± 0.01, *p* < 0.0001) and the number of painful joints (regression coefficient ± *SE*: −0.022 ± 0.0029, *p* < 0.0001) decreased significantly throughout the 3 months treatment period. NSAIDs intake and topical drug application were significantly decreased by 64% (*p* < 0.0001) and 79% (*p* < 0.0001) after 12 weeks, respectively. Only 3/239 (1.3%) patients reported side effects probably related to the product. 80.3% were satisfied with the treatment and 75.5% wished to continue treatment.

**Conclusion:**

This is the first clinical trial showing that *C. longa* and *B. serrata* resin can relieve symptoms in patients with hand osteoarthritis. The study provides useful information for the design of a clinical trial including a broader population.

## Introduction

Osteoarthritis (OA) of the hand is a common musculoskeletal disorder, with prevalence rising steeply with increasing age. The aging-related hand discomfort is associated with pain, stiffness, functional limitation, decreased grip strength, and reduced quality of life. Clinical hallmarks of hand OA include bony enlargement and deformities of the hand joints, at times accompanied by soft tissue swelling (Marshall et al. 2018; Plotz et al. 2021).

There have been a growing number of clinical trials investigating pharmacological and non-pharmacological treatments on hand OA (Marshall et al. 2018; Persson et al. 2020; Whittaker et al. 2021). Based on these recent studies, the European League Against Rheumatism (EULAR) has updated its guidelines specific for hand OA management. The recommended pharmacological modalities included topical treatments preferred over systemic treatments based on safety reasons (topical and NSAIDs being first-line choice), oral analgesics (particularly NSAIDs to be considered for symptom relief for a limited duration), chondroitin sulphate (for symptom relief), and intra-articular glucocorticoids (generally not recommended, considered for painful interphalangeal OA). Clearly, there is now a growing need for supportive therapy to standard care for managing hand OA symptoms (Kloppenburg et al. 2019).

Recently, particular attention has been given to *Curcuma longa* L. (Zingiberaceae) and *Boswellia serrata* Roxb. (Burseraceae) extracts. The efficacy and safety of these extracts have already been established in several clinical trials and are known to have potent anti-inflammatory and analgesic effects in chronic conditions such as osteoarthritis (Bannuru et al. 2018). Curcumin (diferuloylmethane) is the principal curcuminoid extracted from the *Curcuma longa* root and is known as a powerful antioxidant. Boswellic acids, the main active ingredients of *Boswellia serrata* gum, are known to inhibit the 5-lipooxygenase (LOX) pathway, which is a primary source of pro-inflammatory leukotrienes. Curcumin and boswellic acids also have inhibitory effects on the nuclear factor κB (NF-κB) signalling pathway and its gene products, some of which are directly involved in the inflammatory processes and connective tissue extracellular matrix degradation (Henrotin and Mobasheri 2018; Kim et al. 2020). Both *Curcuma longa* and *Boswellia* formulations have been shown to counteract decreases in glycosaminoglycan levels and impede the secretion and activity of matrix Metalloproteases (MMPs), which could potentially forestall further degradation of connective tissue. In summary, many *in vitro* studies suggest that *Curcuma longa* and *Boswellia serrata* could provide a therapeutic benefit that extends beyond symptom relief to disease modification. Two recent meta-analyses evaluating a large number of dietary supplements ranked *Curcuma longa* and *Boswellia serrata* among the most effective compounds for pain reduction in OA at the short term although the quality of evidence was low (Bannuru et al. 2018; Liu et al. 2018). Altogether, these elements give a good rationale to combine *Curcuma longa* and *Boswellia serrata* to relieve joint discomfort in hand OA.

This clinical study was designed to explore in a real-life general practice setting the effects of a combination of *Curcuma longa* and *Boswellia serrata* extracts (CBE) on the algo-functional status of a large sample of patients suffering from hand pain. To the best of our knowledge, it is the first study clinical investigation of this formulation in patients suffering from hand discomfort.

## Materials and methods

### Study design

This is a non-controlled, non-randomised, post-observational clinical study based on a random sample of Belgian General Practitioners (GP) dealing with patients with chronic hand pain. For each GP, patients were recruited sequentially and not selectively. Patients were evaluated at study entry (baseline) and after respectively 6 weeks and 3 months of treatment with CBE (specific bioactive *Curcuma longa* extract CURTIL02, *Boswellia serrata* resin, and vitamin D) as prescribed by their physician in addition to the standard treatment. Patients had to pay for the CBE cure. All demographic patient characteristics and clinical outcomes were collated on a paper ‘case report form (CRF)’ by the attending physician. According to the Belgian Law of 7 May 2004 on human experiments, seeking approval by an ethical committee for a post-doc observational study of data collected from the records of general practitioners (GPs) was not required. Moreover, the dietary supplement is taken in accordance with good medical practice, without the patient’s assignment to a given therapeutic strategy, as the decision to take the product, already on the market and available without prescription in Belgium, was not related to the study and did not require additional diagnostic or monitoring procedures. The study was recorded on ClinicalTrials.gov with the identifier NCT05089318.

### Population

The study involved 239 patients followed by their GP between 1 September 2019, and 1 September 2021. All the patients suffered from chronic hand pain. A total of 46 GPs contributed to the recruitment of patients (5 ± 2 patients per GP).

### Data collected

For each patient the following data were collected: demographics, location, cause and history of pain, type of pain (‘at rest’, ‘when moving’, or ‘all the time’), analog visual scale (VAS) assessment of pain experienced during the last 48 h, number of painful joints, the pain-related limitation in usual movements (functional limitation) evaluated on a VAS (0 = no limitation to 10 = extreme limitation), treatment history and treatment used during the follow-up. Patient satisfaction was assessed on a 5-point grid (from ‘very unsatisfied’ to ‘very satisfied’). The occurrence of adverse effects was specified by the patient and noted by the physician.

### Treatment

Each patient received three-month treatment with CBE. The posology prescribed by the physician was 2 caps at breakfast and 2 caps at dinner for three months. The tested CBE (specific bioactive *Curcuma longa* extract CURTIL02, *Boswellia serrata* resin, and vitamin D named Flexofytol^®^PLUS) was a pharmaceutical-grade food supplement that has received approval from Belgian competent authorities (NUT/PL31/145; Federal Public Service, Health, Food chain safety, and environment) and was commercialised by Tilman SA (Baillonville, Belgium). The HPLC fingerprint and the Certificate of Analysis of the curcumin batch contained in the tested product were provided as supplementary files (Supplementary files 1 and 2). Each capsule contained 89 mg of bio-optimized turmeric rhizome extract (*Curcuma longa* L.) containing 72 mg of curcumin and 120 mg of *Boswellia serrata* resin containing 78 mg boswellic acids and 1.8 µg vitamin D corresponding to 72 UI. For rescue analgesia, patients were allowed to maintain their treatments as long as necessary, including the use of paracetamol and NSAIDs (oral or local).

### Statistical analysis

The statistical analysis was carried out on all eligible intention-to-treat patients enrolled in the study. Only patients with a history of treatment with a curcumin food supplement, patients who received concomitant treatments with Symptomatic Slow Acting Drugs (SYSADOA), corticosteroids, or other turmeric-based products during the study, as well as patients for whom no evaluation of the pain was recorded, were excluded from the study. Results were expressed as mean and standard deviation (*SD*) or as the median and interquartile range (IQR) for quantitative variables and as frequency tables for categorical variables. The numbers of areas affected in the left and right hands were compared by Student’s paired *t*-test. Scores before and after treatment were compared by Wilcoxon signed-rank test for paired observations. The McNemar test was used to compare paired proportions. Linear mixed-effects models were used to analyse the evolution of pain scores over time and to test the effect of patient baseline characteristics on treatment response. Ordinal logistic regression was used to assess the impact of patient and pain characteristics on treatment satisfaction. Results were expressed as regression coefficients with their standard error (SE) Statistical calculations were always based on the maximum number of observations available. Missing values were not replaced or imputed. Results were considered significant at the 5% level (*p* < 0.05). All analyses were performed using SAS statistical software version 9.4 (SAS Institute, NC), and R version 3.6.1.

## Results

### Demographic data

Of the 239 subjects recruited, two subjects were excluded because they took the CBE, four because they took concomitant treatments during the study, and one because the pain was not recorded, yielding a total of eligible 232 subjects for the statistical analysis. Their mean age was 66 ± 12 years and there were predominantly women (75.6%). At baseline, the pain over the past 48 h averaged 6.4 ± 1.6 cm on the VAS scale. The total number of painful joints (left and right hands) was on average 6.1 ± 5.4 out of a total of 30 screened joints. There was no statistically significant difference between the number of painful joints on the left and right hands (*p* = 0.26). About half (49%) of the subjects presented with a deformity of the fingers and in the majority of cases (82%), the pain was more intense on movement. The median pain duration was 12 months (IQR: 6–14 years) (Table 1).

**Table 1.** Patients baseline characteristics (*N* = 232 patients).

		Mean ± *SD* or		
Variable	*N*	Number (%)	Range	
Age (years)	224	66 ± 12	22–98	
Female gender	226	164 (75.6)		
Last 48 h pain intensity (0–10)	232	6.4 ± 1.6	2–10	
Number of painful joints				
Left hand (/15)	232	3 ± 2.9	0–15	
Right hand (/15)	232	3.1 ± 3	0–15	
Total (/30)	232	6.1 ± 5.4	1–30	
Joint deformity	230	113 (49)		
Pain history (months)*	202	12 ± 10	1–40	
Pain increased by movement	228	188 (82.5)		

*Median 12 (IQR: 6–14) months.

### Treatment history

Treatment was initiated according to the recommended dosage (daily intake of 4 capsules, 2 in the morning with breakfast and 2 in the evening with a meal, for the first 6 weeks) in 201 patients (86.6%). For 29 patients another dosage was prescribed (2 × 1 capsules: *N* = 12, or 1 × 1: *N* = 1) or the treatment was modified (2 × 2 then 2 × 1 capsules) during the first 6 weeks (*N* = 16). Among the 216 patients who attended the follow-up visits at 6 weeks and 3 months, 154 (71.3%) followed the recommended dosage throughout the study period and 47 (21.8%) didn’t have any concomitant treatment.

## Treatment effect

### Pain

**Figure 1.**
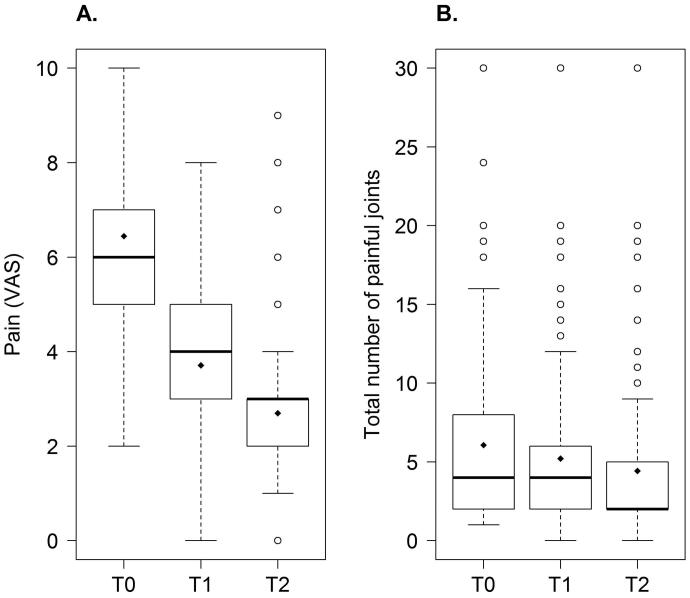
Effect of 3-month CBE treatment on pain scores (A) and on a number of painful joint joints (B) during the last 48 h preceding assessment. A significant difference between baseline and treatment was observed with both *p*-values <0.0001. Boxplots were reported at Baseline (T0), at 6 weeks (T1), and 3 months (T2).

Pain scores decreased significantly (regression coefficient ± *SE*: −0.19 ± 0.01, *p* < 0.0001) throughout the treatment period (Figure 1(A)) and so did the number of painful joints (−0.022 ± 0.0029, *p* < 0.0001) (Figure 1(B)).

### Factors influencing treatment responses

Linear mixed-effects modelling of the time-related data showed that sex and age did not influence the effect of CBE treatment on pain. Similarly, the presence of finger deformities or more intense pain on movement as well as pain duration had no impact on the CBE treatment effect. By contrast, a significant interaction was found between pain intensity at baseline and treatment effect, so that the higher the pain level at baseline, the more important was pain relief at the end of the treatment (*p* = 0.0010). The number of painful joints had also a negative impact on response to treatment (*p* = 0.036). Patients taking any concomitant drugs (NSAIDs, painkillers, topical) had similar CBE treatment efficacy as the others (*p* = 0.61) (Table 2).

**Table 2.** Factors influencing the treatment effect on pain as derived by linear mixed effects model analysis applied to time-related VAS pain scores (*N* = 189).

Covariate	Coef. ± *SE*	*p*-Value
Intercept	0.34 ± 0.52	–
Age (years)	0.009 ± 0.005	0.095
Female gender	−0.16 ± 0.14	0.25
Pain at baseline	0.71 ± 0.057	<0.0001
Number of painful joints	0.031 ± 0.015	0.036
Finger deformities	−0.19 ± 0.13	0.17
Pain duration (months)	0.0059 ± 0.0065	0.37
Pain increased during motion	0.099 ± 0.17	0.55
NSAIDs baseline	0.16 ± 0.13	0.20
Pain killer baseline	0.043 ± 0.13	0.73
Topical baseline	−0.055 ± 0.13	0.67
Time (weeks)	−0.082 ± 0.036	0.023
Pain at baseline × time	−0.017 ± 0.0052	0.0010

## Concomitant treatments

As seen in Table 3, there was a significant decrease over time of NSAIDs of 64% after 12 weeks (*p* < 0.0001) and a significant decrease of topical drugs of 79% (*p* < 0.0001) but for painkillers only a tendency was noted (*p* = 0.083). The percentage of patients taking at least one concomitant treatment fell from 86.7% at baseline to 59.6% at a 3-month visit (*p* < 0.0001).

**Table 3.** Distribution of concomitant treatments (*N* = 232 patients).

Type of treatment	Baseline* (*N* = 232) Number (%)	6-week (*N* = 230) Number (%)	3-month (*N* = 218) Number (%)	*p*-Value
NSAIDS	128 (55.2)	64 (27.8)	46 (21.1)	<0.0001
Pain killers	150 (64.7)	115 (50.0)	91 (41.7)	0.083
Topical	71 (30.6)	30 (13.0)	15 (6.9)	<0.0001
Others	17 (7.3)	11 (4.8)	10 (4.6)	

*Reported in patient history.

### Patient satisfaction

More than 80% of the patients were satisfied or very satisfied with the treatment, 11.9% were neutral and 5.5% were dissatisfied. Satisfaction was neither associated with age and sex, nor with initial pain characteristics of the patient (pain at baseline, finger deformities, pain duration, the pain increased during motion) (all *p* > 0.05).

At the end of the 3-month treatment period, 158 (72.5%) wished to continue the treatment, and 37 (17%) did not want to continue. No patient feature or baseline pain characteristic could be identified to explain the desire to continue treatment.

### Adverse events

After 6 weeks of treatment, 13 (5.7%) patients reported adverse effects. These included primarily diarrhoea (*N* = 4) and stomach pain (*N* = 4). Other effects were nausea (*N* = 1), transit and gastric disorders (*N* = 2), constipation (*N* = 1), or undescribed (*N* = 1). After 3 months of treatment, only 3 adverse effects were reported, diarrhoea (*N* = 2) and undescribed (*N* = 1).

## Discussion

This open-label study showed that the combination of *Curcuma longa* and *Boswellia serrata* extracts decreased rapidly and significantly chronic pain among subjects with hand OA. Pain relief was already significant after 6 weeks of dietary supplementation and the effect continued to increase with treatment duration. The dietary supplement decreased pain intensity by 30% or more in 89% of the patients. Effect size (ES) on pain VAS, calculated as the mean difference from baseline to endpoint divided by the standard deviation, was 2.1 which is over twice the placebo effect. In RCT, the effect size of placebo in hand osteoarthritis pain is 0.80 (Zhang 2019).

The CBE treatment response was positively affected by pain intensity at baseline, while sex, age, joint deformities, or NSAIDs intake did not affect it. In other words, CBE was more efficient in the most painful subject, suggesting that CBE could be particularly prescribed to manage, in combination with standard treatments, discomfort during flare-ups of chronic conditions such as OA (Haroyan et al. 2018; Liu et al. 2020).

The ability of boswellic (*Boswellia serrata*) and turmeric (*Curcuma longa*) extracts (alone or in combination) to relieve patients with knee OA has been largely described in the literature (Bannuru et al. 2018; Liu et al. 2018). However, to the best of our knowledge, this is the first and unique study reporting the effect of this combination in hand OA. Recently, an internet-based, parallel, randomised, placebo-controlled trial concluded that the combination of proprietary products containing *Boswellia serrata* extract (Boswellin Super), pine bark extract (Fenoprolic 70 organic), and methylsulfonylmethane and curcumin (Flexofytol) had no effect on symptomatic relief in people with symptomatic hand OA (Liu et al. 2021). A number of factors may explain the absence of the effect of these compounds. Specifically, the study included a majority of patients with a severe radiological form of hand OA among which more than one-third had erosive OA. In contrast, our study included patients with chronic pain associated with OA condition diagnosed by general practitioners without any radiological confirmation of diagnosis. The second difference is the method used to collect the study data. In the Liu et al. (2021) study, data were collected online while in our study the data were collected by the physician. This methodological difference is important since it is well-known that in online studies there is a majority of less technology confident participants. In the Liu et al. (2021) study, there were fewer technology confident participants in the supplement combination group (54%) than in the placebo group (70%). Further, these authors demonstrated that participants with greater technology confidence demonstrated more pain relief with their mixture than those who were not. Finally, the dosage used in the Liu et al. (2021) study was below the usual manufacturer-recommended dosage. For example, they used one capsule of Flexofytol corresponding to 46.6 mg of bio-optimized *Curcuma longa* extracts while the evidenced efficient dose in knee OA was 186.6 mg (Henrotin et al. 2019).

This study presented some limitations, the main one being the absence of a control group. Therefore, the effects of the combination tested might have been overestimated. The promising results observed in this preliminary study will have to be confirmed in a controlled study. The hand OA is grossly characterised, thus limiting data interpretation. For example, the ratio of patients with erosive hand OA is unknown.

## Conclusions

Pharmaceutical grade CBE at a prescribed dose of two tablets twice a day and in support of standard treatment was able to significantly reduce hand chronic pain. This effect, more important in the most painful subjects but not associated with NSAIDs intake, suggests that CBE could be used in complement to standard care to manage flare in disease conditions like OA. Together with a significant reduction of concomitant NSAIDs intake and good tolerability, these findings also indicate that CBE is potential support for the treatment of hand joint discomfort. Finally, the study provides useful information for the design of a larger phase III clinical trial including the sample size estimate, the choice of the dose, and the selection of primary outcomes
